# Geriatric Interprofessional Simulation Education Results and Rapid Cycle Quality Improvement

**DOI:** 10.1093/geroni/igab046.004

**Published:** 2021-12-17

**Authors:** Denise Kropp

**Affiliations:** Northeast Ohio Medical University, Rootstown, Ohio, United States

## Abstract

Following a geriatric interprofessional education event, we measured learner progression in interprofessional collaborative competencies using the Interprofessional Socialization and Valuing Scale (ISVS). We also measured student satisfaction with an investigator generated assessment tool. Through Rapid Cycle Quality Improvement (RCQI) processes, we implemented a number of variations of both the in-person and the virtual events. Variations included differences in case studies, pre work requirements, geriatric didactic topics, poster topics and presentation format, facilitator training, standardized patient or patient presence, huddle format, and demonstration of how to effectively perform teamwork. Results showed gains in interprofessional collaborative competencies between pre- and post-education using this geriatric simulation model. Learner satisfaction was high for all simulation variations. Results of education variations and comparisons of the delivery methods will be presented.

